# What can healthcare systems learn from looking at tensions in innovation processes? A systematic literature review

**DOI:** 10.1186/s12913-022-08626-7

**Published:** 2022-10-28

**Authors:** Malte Haring, Felix Freigang, Volker Amelung, Martin Gersch

**Affiliations:** 1 Hannover Medical School, Institute of Epidemiology, Social Medicine and Health System Research, Carl- Neuberg-Str. 1, 30625 Hannover, Germany; 2 inav – private institute for applied health services research GmbH, Schiffbauerdamm 12, 10117 Berlin, Germany; 3 Department of Information Systems, Freie Universität Berlin, Garystr. 21, 14195 Berlin, Germany

**Keywords:** Healthcare system, System innovation, Innovation, Process, Tensions, Systematic literature review

## Abstract

**Background:**

Until now, scholarship on innovation processes in healthcare systems lack an in-depth appreciation of tensions. Tensions often revolve around barriers and result from individual assessments and prioritizations that guide actions to eventually overcome these barriers. In order to develop a more differentiated understanding of tensions’ role in healthcare innovation processes, this paper aims to shed light on the multifaceted ways in which tensions emerge, are being dealt with, and how they hinder or, at times, facilitate innovation processes.

**Methods:**

A systematic review of published and grey literature was conducted following the Preferred Reporting Items for Systematic Reviews and Meta-analyses (PRISMA) guideline. The review involved searching three databases for original research articles and manually searching citations. Twenty-nine original full texts were identified, evaluated, and coded. These include papers on innovation in healthcare systems that investigated innovation-related organizational tensions. The findings were synthesized into different types of tensions in healthcare system innovation and the descriptions of the conflicting elements. We also analyzed the investigated innovations by type, process stages, and across different countries and healthcare systems.

**Results:**

A total of forty-two tensions were identified and grouped into nine categories. Organizing tensions were predominant, followed by learning/belonging, performing, and performing/organizing tensions. Tensions most frequently occurred in the implementation phase and in the form of a dilemma. Included studies were conducted mainly in government-funded healthcare systems.

**Conclusion:**

Our data suggest that innovation processes in healthcare systems are impaired by conflicts between contradictory elements, working cultures, and convictions and the organizational and regulatory context. Since the majority of the tensions we collected in our study can be addressed, future policy-making and research should take advantage of this fact and develop strategies that significantly influence the successful management of tensions and thus improve the implementation of innovations.

## Introduction

Introducing innovation into a healthcare system’s primary care market remains a challenging task. Even though healthcare systems differ in governmental type and arrangements, the challenge to overcome barriers in innovation processes is common to all stakeholders who aim to design and shape effective healthcare services. Various factors are responsible for this: Different legal, economic and organizational structures have to be considered when integrating innovations; national systems are also characterized by strong institutionalization, regulatory challenges and process complexity [1–3].

In addition, the stakeholders involved may have different views on innovation and the associated economic effects, and the implications for the organizational and ethical orientation of care delivery [4]. This is also expressed by the different values and perceptions that characterize the heterogeneous working cultures and stakeholder groups in healthcare. Just as different characteristics of community, control, cure, and care shape the treatment process itself, the confrontation between working cultures and groups has a general impact on the development of healthcare [5].

The fact that there are a multitude of contradictory and rarely aligned elements, stakeholders, and working cultures, each with their own needs and interests, increases the complexity of the innovation process [1, 6]. As a result, healthcare innovation is influenced by the framework conditions and inertia of the system, while simultaneously being driven by an openness for new developments among the stakeholders. This systemic constraint hinders the transfer of innovative approaches to healthcare systems and the implementation of innovations in the primary healthcare market [2, 3, 6].

Therefore, a better understanding of these multi-layered and sometimes resistant structures, relations and processes that determine the path of an innovation from invention and development to deployment and dissemination in the healthcare system is needed [1, 7]. The path of innovation is a sequence of decisions, reactions, and events. Although usually idealized as predictable and, ideally, frictionless, it involves different stakeholders with various, sometimes competing demands and, as such, is repeatedly characterized by tensions [8–11]. On the one hand, these tensions are a symptom of possibly avoidable difficulties in the introduction of innovations. On the other hand, they have the potential to precipitate actions and reactions. Organizational reflexivity is required to steer the overarching innovation process and prevent unintended changes of course. Successful innovation is therefore a balancing act between strategic foresight, necessary agility and “muddling through” [12–15].

Tensions have already been discussed in other organizational settings, e.g., inter-professional collaboration, digital platforms, or project management [16–18]. However, in healthcare research, scholars have mainly focused on examining (systemic, technological and/or organizational) barriers to describe the challenges of healthcare innovation [2–4, 19, 20]. While being critiqued for its conceptual fuzziness, a barrier – as a heuristic device – also tends to suggest a rather static condition and therefore does not allow to fully grasp the dynamic processes involved in dealing with obstacles in healthcare innovation [21]. The concept of tensions, on the other hand, is process-sensitive and explicitly addresses the relational aspects of maneuvering conflicts and challenges. We argue, that the concept of tensions is better suited to make sense of the complex ways in which healthcare innovation can be accomplished [22].

In order to ground the concept of tensions in empirical research and to better understand its role in the implementation of healthcare innovations, a systematic review of the literature on tensions in healthcare innovation has been conducted. The review was guided by the following questions: What tensions have been previously assessed in the context of innovation in health care systems? What lessons can decision makers in health systems learn from looking at tensions in innovation processes? We focused on tensions and trajectories related to innovation processes and explored their underlying causes. Furthermore, our goal was to gain insights into the successful management of innovation processes and to integrate the corresponding implications for healthcare practice to enable more control and to improve the outcomes of healthcare innovation processes. By reviewing existing research on innovation in healthcare and recommendations for action in health systems design, we aimed to uncover, analyze, and categorize typical, interrelated tensions that evolve during the innovation process in healthcare systems.

We identified the types of tensions that typically emerge, the individual conflicts that cause them and the systemic mechanisms underlying them. To enable a process-sensitive analysis, we classified the identified tensions into different categories by type (e.g., organizing, performing, learning, and belonging) as proposed by Smith and Lewis and by phase of the innovation process in which they occur [22, 23]. We additionally examined whether there are other healthcare-specific categories of tensions than the aforementioned.

With these descriptions and classifications, we provide a typology of tensions in healthcare system innovation that might serve as a starting point for the creation of sufficient strategic framing and an improved development of innovation processes based on a better understanding of the dynamics in innovation processes, the importance and effect of individual behaviors and the underlying causes of tensions.

## Conceptual foundations

### Innovation in healthcare systems

Innovation can be both the outcome of a process and the process from which innovation emerges. In our study, we focus on the innovation process with the understanding that it is a nonlinear sequence of practices and examine how they are interconnected over space and time through actions, reactions, and events as well as a constant interplay of technology, organization, and people on both the supply and demand sides. On the supply side, the term usually encompasses the invention, development, and innovation process to market entry. On the demand side, it is then the acceptance and perceived uncertainty on the market. Driven by technical or organizational innovation, the outcome of (successful) innovation processes can represent, for example, process innovation, institutional innovation, or innovative structures [9, 24–26].

The evaluation of successful innovation is usually carried out according to an improvement such as “long-term sustainable growth” [24 p 226]. In addition to this consideration of the complexity, diversity, and variability of the innovation process, scholars have enriched the discussion of innovation by taking into account the fact that different actors with different capabilities and interests are involved [8, 27, 28].

These various elements can also be observed in healthcare. Due to the specifics of healthcare services and the threat of market failure, national regulations for the primary healthcare market vary around the world. Each nation has its own framework for innovation processes that applies for the particular healthcare system respectively the organizations, stakeholders and procedures included in it [3, 29]. The resistant structures and mechanisms have great forces of inertia, and innovations that are not mapped in these mechanisms encounter massive structural barriers and resistance to change. Innovation researchers refer to this as a lock-in of the healthcare system [21, 30].

Healthcare system improvement requires multi-level action and a quadruple aim approach: enhancing the patient experience, improving the health of the population, improving the working conditions of healthcare providers, and lowering the per capita costs of healthcare [31]. This is also illustrated by the increasing discussion about value-based healthcare and the optimization of patient benefit under economic conditions [32]. Thus, implemented innovation activities and approaches should be ideas and practices that are proven effective in achieving these goals. Aiming at increasing and improving the standards and efficiency of a healthcare system, healthcare system innovations encompass a wide range of elements, from process efficiency to treatment to research. A distinctive feature of the health sector is that added value does not necessarily have to be an economic or efficiency improvement, but can also translate into increased quality, safety or better outcomes for patients, stakeholders, or the healthcare system in general [33].

While some healthcare systems, especially those of Western countries, face major demographic, economic and political challenges, a wide range of innovations and responses to these can be observed [1, 3]. These include technological, product, process, organizational, and service innovations. Innovative management approaches aimed at business models, research and development processes are also being pursued at the regulatory level [1, 33]. Attempts to translate these elements into a healthcare system frequently end up with small projects limited in time or region. In addition to the characteristics of the healthcare system, the change process is interlinked, and the involved stakeholders as well as their tasks and roles vary and change over time and in different situations [33].

When innovation is not viewed as an object that mechanically passes through a sequence of linear stages, the dependence on the context in which the innovation is designed and created becomes significant [34]. Individual actors (micro level), projects and teams (meso level), and the wider circumstances, regulations, and frameworks (macro level) have multiple, divergent, and conflicting skills, interests, and perspectives. This entails correspondingly divergent speeds and directions of organizational, legal, and technical change. The complexity is further increased by contradictory mechanisms and funding, accountability, patient/customer-related factors, and technology use. The interplay and combination of these variables has a substantial effect on the development and implementation of an innovation into a healthcare system [2, 8, 33]. Stakeholders and managers have to deal with this complexity and adapt their strategy to the specific context while at the same time managing tensions that may arise between the different perspectives and competing demands involved in the innovation process [10].

### Trajectories, tensions, and management strategies

Innovation processes are often shaped by conflicts, competing demands, contradictions, dilemmas, and tensions [35]. To further the understanding and discussion of innovation process, innovation scholars have recognized the importance of event patterns. In particular, the temporal order of events and the interactions of different entities are relevant factors that impact the outcome of a process [36, 37].

When tracing patterns in the unstructured mass of process data, the challenge is that events in an innovation process do not occur in predictably linear and sequential stages [34] and that the involved entities are difficult to study in isolation. Recent research has developed the strategy of “temporal bracketing” of the process to address this problem. To gain insight into actions and changes along the development path, an innovation process is divided into several analytically relevant episodes. This process is called bracketing and enables the analysis of data (actions, reactions, events, situational assessments and so on) within a given period as well as correlations and linkages between actions of temporally separated episodes [38].

The perspective on the path from the development of an idea to its implementation is understood as the trajectory dynamics of innovation [8, 11]. Along the innovation trajectory, tensions arise when apparent polarities and divergent perspectives, constructed by individual actors, conceal the simultaneity of contradictory elements [10, 22, 39]. Tensions can then serve as empirically ascertainable perceptions of problems of different actors and as explanations for their actions: Depending on the occasion, contradictions become apparent over time, and actors perceive the different elements of tension to be of varying importance or threat. This leads to (re-) actions, which continue the sequence of action, reaction, and event [13, 15, 18]. “*Innovation processes are riddled with tensions*” [14 p463] as actors have to make these decisions constantly. Interaction of the different groups or individuals and discussion of the varying interests is required to reconcile these tensions. The agreements negotiated and reached in this way establish how to proceed and determine the further trajectory of innovation [12].

Contradictions become salient for individuals dealing with tensions. Scholars have recognized coexisting contradictions in a wide range of research fields and in diverse organizations and settings. Consequently, different types, terms, and constructs of contradictions are associated with tensions. Dilemmas and paradoxes are relevant examples. In the present study, we classified these under the umbrella term “tensions” [22, 40, 41].

Dilemma is a type of tension in which each element or pole has clear advantages and disadvantages [22]. One example of a dilemma in the modernization and digitalization of today’s healthcare systems is the tension between the use and protection of patient data when it comes to the implementation and use of electronic health records. This dilemma is fueled by unresolved ethical, financial, and legal conflicts that impair the harnessing of innovation in healthcare systems [42]. Conflicts between the elements of such dilemmas can potentially be resolved. To enable this, the advantages and disadvantages of the elements need to be weighted so that an “either/or” decision or a trade-off can be made [22, 40, 41]. A dilemma becomes paradoxical when these trade-offs persist and/or re-emerge over time. Smith and Lewis define paradoxes as “*contradictory yet interrelated elements (dualities) that exist simultaneously and persist over time; such elements seem logical when considered in isolation, but irrational, inconsistent, and absurd when juxtaposed*” [22 p387]. Paradoxical tensions may be nested or interwoven, and the contradictory demands are in an ongoing, changing interaction. They consist of the underlying tension between the elements and the responses of a decision maker addressing them. A “real” paradox occurs only in very rare cases [22, 43]. An example that illustrates these principles of a paradox and thus the difference from a potentially resolvable dilemma is that of the liar paradox: “This statement is false”. There is no way to resolve the contradictions, because there is no way for the statement to be true and false at the same time [44].

Effective management of paradoxical tensions attends both poles and gives decisive and cyclical responses. It may spur virtuous cycles [41, 45, 46] and lead to more positive tensions and synergies that promote innovation in the further trajectory [13, 47]. On the other hand, failing to address paradoxical tensions leads to vicious cycles and downward loops. Decision makers who emphasize one pole of the paradox more than the other intensify counter-demands and opposing positions and effect limited outcomes [39, 40]. Shifting from vicious to virtuous cycles demands managing actions so as to respond to and cope with tensions [17].

Three general management strategies to spur virtuous cycles and avoid vicious cycles are the either/or, both/and, and more-than approaches [13, 18, 22]. Either/or leadership seeks to separate contradictory demands and resolving the conflict by choosing one element over the other or by separating tensions in space and/or time [13, 18]. This choice can also fuel vicious circles in some cases [39]. While dilemmas contain the potential for resolution, paradoxical tensions are characterized by interdependency and persistence, and demand a more elaborate management strategy than either/or [40]. Both/and approaches to contradictory elements seek to achieve dynamic equilibrium or balance between and integration of the two poles while considering the interdependence of the conflicting elements. However, this leadership approach may trigger vicious cycles and prevent organizations from tapping the positive potential of tensions [13, 22]. More-than leadership pursues the goal of shaping a new perspective or creating a new element that outperforms the existing elements [13]. The approach reflects that paradoxical tensions may *“seem logical individually but inconsistent and even absurd when juxtaposed*” [22 p382]. Moreover, it synergistically blends two seemingly paradoxical poles of tension by encircling them with each other [48]. When effectively executed, a more-than strategy can tap the positive potential of paradoxical tensions, but synergistic potentials may remain unused [13].

Following Smith and Lewis [22], four main types of organizational tensions have been identified in the management and change literature. *Organizing tensions* arise when organizations generate competing designs and processes, *performing tensions* become salient when a variety of different individuals/stakeholders pursue conflicting goals, *learning tensions* emerge from the renewal or destruction of past practices to create new approaches, and *belonging tensions* surface when individuals/stakeholders identify with competing identities. Six further categories were used to classify cross-category tensions that do not clearly fall within one category but lie between two different categories. Cross-category *learning/belonging tensions* surface when a person’s developed sense of self and purpose and the desire to hold on to it clash when a situation requires adaptation and change. *Tensions between learning and organizing* emerge when operational routines and efficiency goals that pursue stability and discipline conflict with the need to obtain innovative results quickly and flexibly. *Tensions between belonging and organizing* appear when there is conflict between collective action and the properties and characteristics of an individual. *Tensions between performing and belonging* arise when the identities of individuals clash with social and occupational demands. *Tensions between performing and organizing* emerge as a result of conflict between means and ends, for example, between high commitment and high performance. *Tensions between learning and performing* arise when the preservation of current capabilities conflicts with the development of capabilities for a later point in time [22, 39, 40].

## Methodology

### Literature search

After conducting initial background research to define the conceptual foundations of this study, we carried out a systematic literature review as recommended in the PRISMA 2020 statement and guided by the associated checklist [49]. The systematic literature review was conducted from May to September 2021 with no time restrictions by two independent researchers (MH and FF), who engaged in a discussion process with other researchers (MG and VA). The databases PubMed, LIVIVO and Web of Science were used for the study. In addition to specific medical databases, a database focusing on management and change literature was chosen due to the interconnection of medicine and management in the context of this research. Our literature search strategy is shown in Table 1. The searches contained terms and keywords related to *healthcare system* (health service system, national health system, etc., as described in Wendt et al. [50]), *innovation* (innovation, innovator, etc.) and *tension* (tension, conflict, contradiction, etc.). Since tensions, conflicts, and related terms often are not directly stated but used implicitly and since there is no standardized nomenclature on this subject a search strategy that takes the most common synonyms and language variances into account is needed to properly capture the literature relevant to this research question. Therefore, we gradually added terms to a more sensitive manual search in Google Scholar and in selected journals and performed a backward search via PubMed using the additional terms to identify further studies. In addition, the reference list of each original article included was analyzed in order to identify other potentially eligible papers.

**Table 1:** Literature search strategy used, for example, to find articles in the Web of Science database

Database	Search criteria
**Web of Science**	You searched for: (AB=(healthcare system OR pluralist health system OR health insurance system OR health service system OR socialized health system OR public assistance OR health insurance OR national health system OR national health service model OR social insurance model OR private insurance model OR national health service model OR social insurance model OR private insurance model OR bismarckian health insurance system OR beveridgean national health service) AND AB=(innovativeness OR innovate OR innovated OR innovates OR innovating OR innovation OR innovation s OR innovational OR innovations OR innovative OR innovator OR innovatively OR innovator s OR innovators) AND AB=(tensions OR tension OR paradox OR paradoxical OR demand OR demands OR demanding OR competing OR competition OR compete OR competes OR conflicts OR conflict OR conflicting OR contradiction OR contradictions OR contradictory OR dilemma OR dilemmas OR differentiating OR differentiate OR duality OR dualities))

### Study selection

**Fig. 1:**
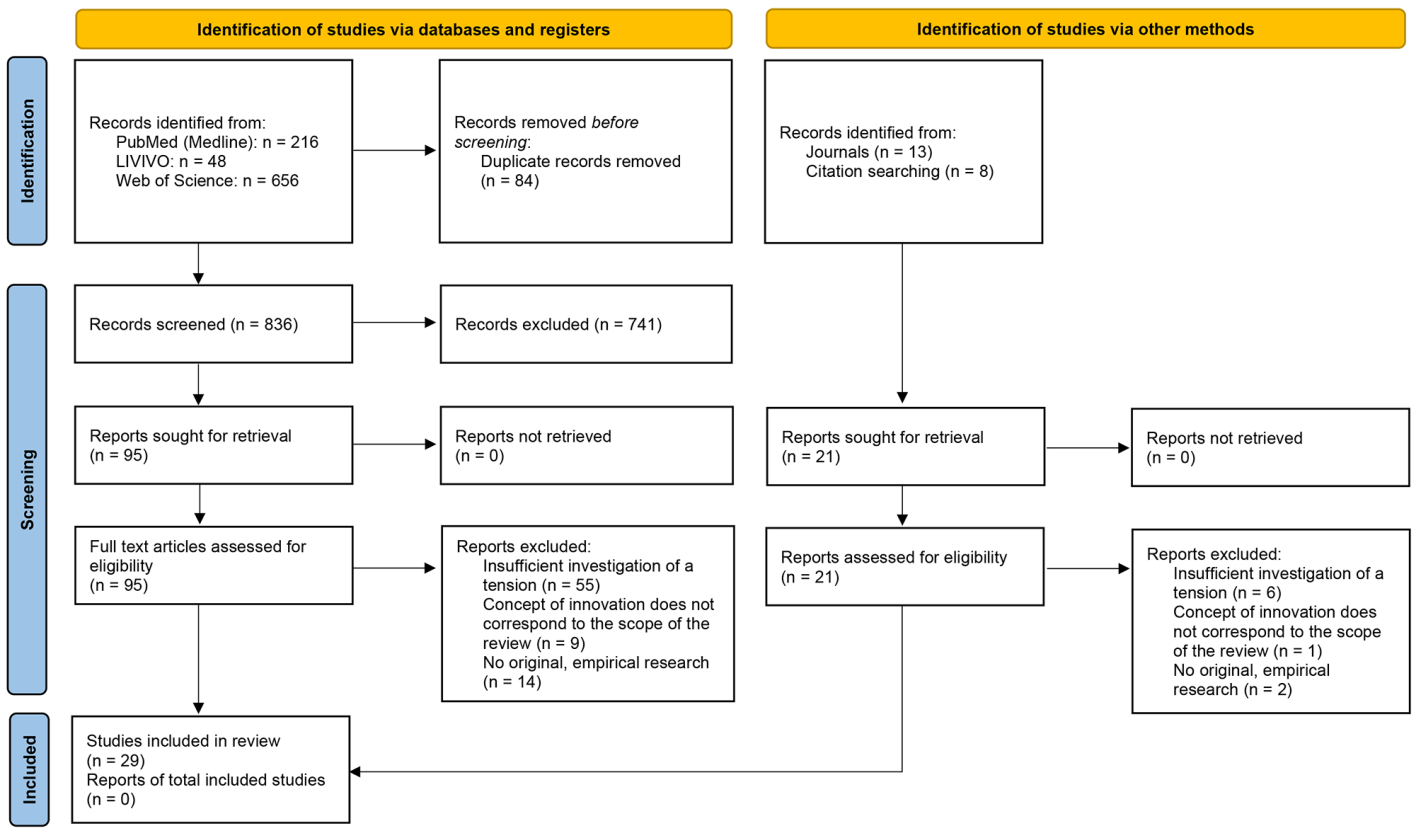
Flow diagram of the systematic literature review process following the recommendations of the PRISMA 2020 statement [49]

The titles, abstracts and full texts of all identified records were screened by two independent investigators (MH and FF). Studies had to meet the following criteria to be included in this review: (a) Language of publication: English or German; (b) object of analysis: healthcare system innovation and (c) detailed investigation of at least one form of organizational tension (based on an inter-rater-oriented assessment of whether the contradictory or conflicting elements under consideration met the definition of tension as described above) in the context of original empirical research. We identified 920 studies in the three databases mentioned above (Fig. 1). Eighty-four duplicates were removed before screening. The titles (n = 836) and abstracts (n = 625) of the remaining records were screened, yielding 95 potentially eligible full-text articles. The full texts were independently assessed for eligibility using the above-mentioned inclusion criteria; included articles thus had to encompass an in-depth, empirical description of at least one tension in the context of healthcare system innovation. This resulted in the selection of 17 articles. Based on the information in these articles, we performed backward and manual searches, which produced an additional 12 articles. Our final literature set consisted of 29 studies of organizational tensions.

### Data extraction, analysis, and classification

Data extraction and analysis was conducted by two reviewers (MH and FF) following an interpretative thematic analysis, in which both reviewers independently read and coded all relevant overarching themes by using a Microsoft Excel database to record, structure and encode information from each study/report. Subsequently, they discussed the results and resolved any coding discrepancies through discussion and consultation with two other researchers (MG and VA).

The coding process encompassed the following steps: The first step was to group the identified organizational tensions into categories as described by Lüscher and Lewis and Smith and Lewis [22, 40]. The reviewers considered whether additional categories were needed to reflect the specific characteristics of a given healthcare systems and innovation. Tensions spanning multiple categories were assigned to the category that best fit the “major” tension discussed in the study, and the minor tensions were noted in the remarks section of the coding table.

The second step was to classify the identified tensions as paradoxes or dilemmas. Since this overlap, this was decided based on the temporal component, in particular the question of whether the described conflict between the opposing, interrelated elements was persistent and/or recurrent and yet unresolved [22]. In the third step, the reviewers considered the process perspective, i.e., evaluated the time course of tensions in innovation implementation using the six-stage Observatory of Public Sector Innovation (OPSI) Innovation Lifecycle proposed by the Organisation for Economic Co-operation and Development (OECD) [23]. Innovation lifecycle analysis helps to understand the cyclical and interconnected nature of the innovation process and the conditions and factors that affect the different stages of innovation. This is crucial to the systematic analysis of organizational tensions such as those that occur in health innovation initiatives and projects. The six stages of the OPSI Innovation Lifecycle are: identifying problems, generating ideas, developing proposals, implementing projects, evaluating projects, and diffusing lessons (i.e., new insights gained). This division of the innovation process into stages also corresponds to the bracketing approach of [38].

The fourth and final step was to evaluate the innovation process over time to better understand the management strategies and tensions related to innovation implementation. The reviewers accomplished this by identifying and evaluating important episodes of each innovation process with regard to the emergence, development, and management of relevant tensions using the either/or, both/and, and more-than approach to the study of organizational dilemmas, paradoxes and tensions [13, 22].

In the next section, we present our findings regarding the type and nature of organizational dilemmas, paradoxes and tensions related to the implementation of innovations across different health systems and nations. To consider both the content and the process, we also recorded the stage at which the identified tensions emerged and classified the tensions as dilemmas or paradoxes according to the categories of Smith and colleagues [15, 22].

## Results

A total of 42 tensions were identified by the reviewers and grouped into nine categories: (1) belonging, (2) belonging/organizing, (3) learning, (4) learning/belonging; (5) learning/organizing, (6) organizing, (7) performing, (8) performing/belonging, and (9) performing/organizing (Table 2). Organizing tensions were the predominant type (n = 10, 24%), followed by learning/belonging (n = 7, 17%), performing (n = 6, 14%), performing/organizing (n = 6, 14%), and belonging tensions (n = 5, 12%).

**Table 2:** Nine categories of tension identified in this review

Category of tension	Number of cases
Belonging	5 (12%)
Belonging/Organizing	2 (5%)
Learning	1 (2%)
Learning/Belonging	7 (17%)
Learning/Organizing	2 (5%)
Organizing	10 (24%)
Performing	6 (14%)
Performing/Belonging	3 (7%)
Performing/Organizing	6 (14%)
**Total**:	**42**

**Fig. 2:**
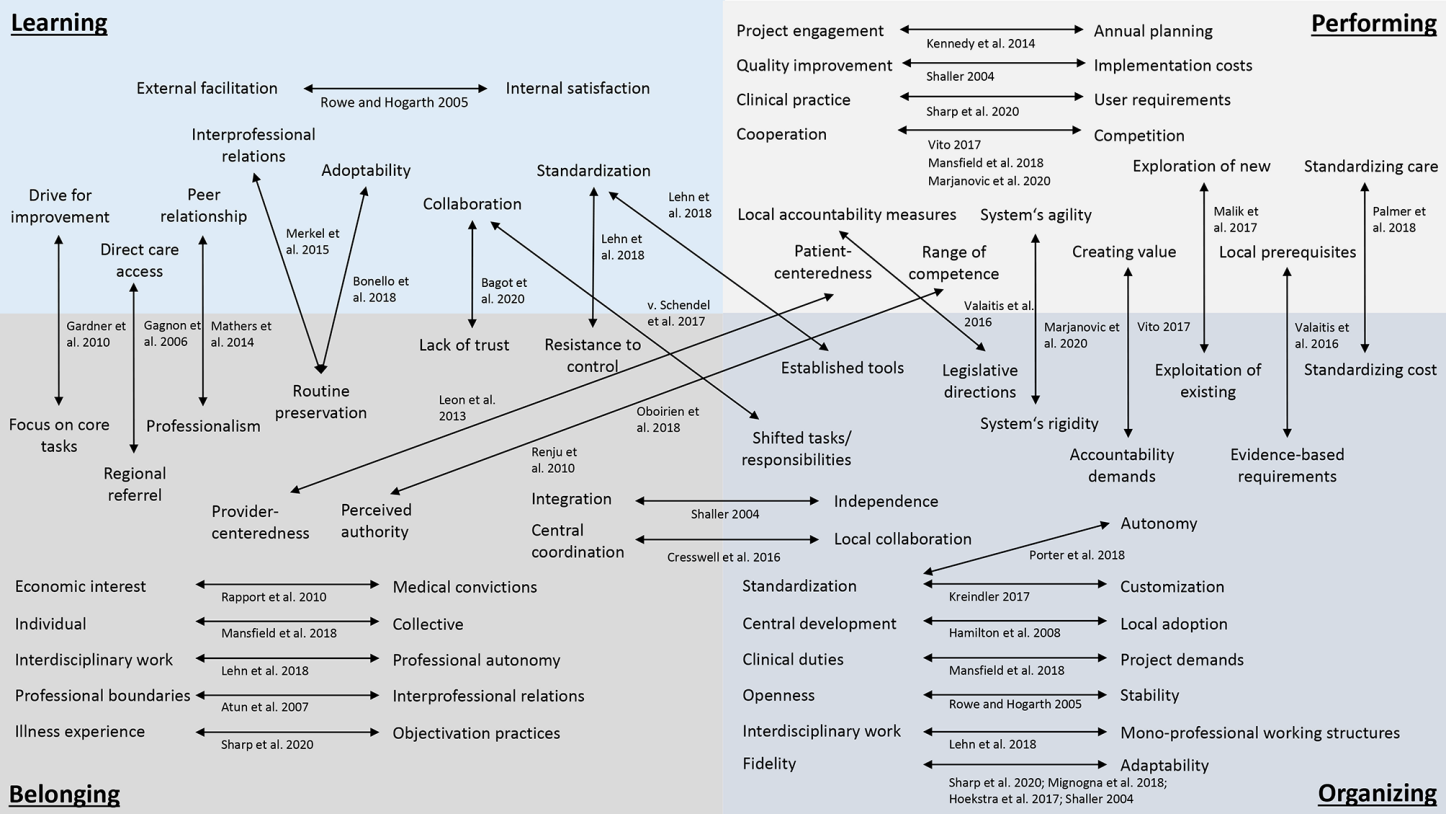
Classification of identified tensions in healthcare innovation based on Mini and Widjaja [18]

The identified tensions are shown in the flowchart in Fig. 2, naming the authors in whose studies the tensions were described. The arrows are used to contrast the contradictions. Depending on the category to which the tensions are assigned, they are placed in one of the boxes of the main categories or, if they are assigned to one of the cross-categories, they are shown across categories.

**Fig. 3:**
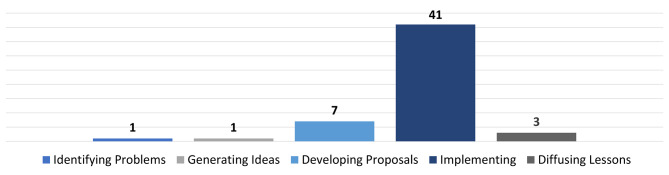
Number of identified tensions in the stages of the innovation process according to OPSI [23]

Approaches such as interviews (n = 25), focus groups (n = 10), case studies (n = 7), and other qualitative survey methods have proven to be effective methods for identifying tensions in healthcare innovation processes that are usually implicitly present or described. The studies were conducted mainly in government-funded healthcare systems. For example, 15 of the included studies were from Beveridge systems in Canada (n = 6) and the UK/England/Wales (n = 9). A further six studies were from the welfare state-organized systems of Australia, Denmark, Germany, and the Netherlands. In contrast, four studies described tensions in the privately organized healthcare system of the United States. Nevertheless, we observed distribution similarities across the different countries and healthcare systems. The stage of occurrence of the innovation-related tensions was also evaluated. Following the definitions of the lifecycle phases of an innovation as described in the OPSI Innovation Lifecycle, tensions most frequently occurred in the “implementing projects” phase (n = 41), followed by the “developing proposals” (n = 7) and the “diffusing lessons” stage (n = 3). One type of tension each occurred during the first two stages of the innovation process, i.e., the “identifying problems” and the “generating ideas” stage. No tensions emerged during the “evaluating projects” phase (Fig. 3). Regarding the classification of the type of tensions we categorized 38 types of tensions as dilemma (91%) and 4 as paradoxes (9%) according to the interpretation of Smith and Lewis [22].

### Distinct categories of tensions

#### Learning tensions

We identified one learning tension using the classification system shown in Table 3. Learning tensions generally result from the renewal or destruction of previous practices in favor of new approaches [22]. One reviewed study describes an example involving conflict between external facilitation and internal satisfaction. Rowe and Hogarth describe this dilemma, which occurred during the implementation of four primary care trust pilot sites. In this specific regional context, the need for external energy to drive things forward and the existing internal comfort and satisfaction were in conflict [51]. During the literature reviews, we identified learning tensions with additional conflicting elements associated; these tensions were not classified as learning tensions but were assigned to the cross-cutting categories *learning/organizing* (Table 4) and *learning/belonging* (Table 5).

**Table 3:** Learning tensions associated with healthcare innovation

Authors	Type of innovation	Stage of innovation process	Type of tension (dilemma or paradox)	Conflicting elements
Rowe and Hogarth (2005)	Four primary care trust pilot sites	Implementing projects	External facilitation vs. Internal satisfaction (Dilemma)	**A: External facilitation**: Need for external energy to challenge the status quo and get ahead (p. 402) **B: Internal satisfaction**: “Comfort zone” of leadership and of the whole organization (p. 402)

**Table 4:** Learning/organizing tensions associated with healthcare innovation

Authors	Type of innovation	Stage of innovation process	Type of tension (dilemma or paradox)	Conflicting elements
Van Schendel et al. (2017)	Non-invasive prenatal testing for foetal aneuploidy	Implementing projects	Collaboration vs. Shifted tasks/ responsibilities (Dilemma)	**A: Collaboration**: Different professional groups working together in a new way (p. 8) **B: Shifted tasks/responsibilities**: Shifting distribution of tasks and responsibilities through the new method (p. 8)
Lehn et al. (2018)	Readmission prevention program	Implementing projects	Standardization vs. Established tools (Dilemma)	**A: Standardization**: Using standardized, digital communication reports to speed up patient enrollment within the program (p. 7) **B: Established tools**: Managing communication according to the established strategy and with the existing tools (p. 7)

**Table 5:** Learning/belonging tensions associated with healthcare innovation

Authors	Type of innovation	Stage of innovation process	Type of tension (dilemma or paradox)	Conflicting elements
Lehn et al. (2018)	Readmission prevention program	Implementing projects	Standardization vs. Resistance to control (Dilemma)	**A: Standardization**: Standardizing a process independent of professional competence to improve the outcome of the program (p. 7) **B: Resistance to control**: Self-conception of a professional to be clinically competent and making decisions based on his/her expert judgement (p. 7)
Bagot et al. (2020)	Acute stroke telemedicine program	Developing proposals, Implementing projects	Lack of trust vs. Collaboration (Dilemma)	**A: Lack of trust**: Lack of trust and confidence in skill between the neurologists and remote hospital clinicians (p. 86) **B: Collaboration**: Increased access to stroke specialists and treatments to eligible patients (p. 80)
Mathers et al. (2014)	One-to-one peer-support from health trainers (HTs)	Implementing projects	Professionalism vs. Peer relationship (Paradox)	**A: Professionalism**: HTS’s capability and validity was challenged by professional NHS staff’s biomedical understanding of therapeutic efficacy (p. 742) **B: Peer relationship**: “HTS’s ‘intervention’ is as therapeutic through its contextual peer relationship as through the delivery of psychological behavior change interventions” (p. 742)
Bonello et al. (2018)	Interprofessional education (IPE)	Implementing projects	Routine preservation vs. Adoptability (Dilemma)	**A: Routine preservation**: Demanding a shift from established norms, behaviors, and paradigms (p. 7) **B: Adoptability**: Creating a collaborative practice-ready workforce (…) to address challenges and improve outcomes across realms and practices (p. 3)
Gagnon et al. (2006)	Telehealth in rural/remote regions	Implementing projects	Regional referral vs. Direct care access (Dilemma)	**A: Regional referral**: Accessing specialized services via the usual referral process to the regional hospital (p. 6) **B: Direct care access**: Accessing specialized services directly via telehealth (p. 6)
Gardner et al. (2010)	Audit and best practice for chronic disease (ABCD) as part of a continuous quality improvement (CQI)	Implementing projects	Drive for improvement vs. Focus on core tasks (Dilemma)	**A: Drive for improvement**: Stimulating improved outcomes and care delivery through staff motivation (p. 7) **B: Focus on core tasks**: Sense of burden due to high demands and uncertainty leads to meeting obligations for core tasks only (p. 7)
Merkel et al. (2015)	Transcatheter aortic valve implantation (TAVI)	Implementing projects	Routine preservation vs. Interprofessional relations (Dilemma)	**A: Routine preservation**: Creating an issue of competence by shifting treatments which originally belonged to cardiac surgeons to the responsibility of cardiologists (p. 6) **B: Interprofessional relations**: Cooperation creates multiple advantages (medical outcomes and the improved quality of life of patients) compared to the standard surgical procedure, thereby supporting the implementation process (p. 5)

#### Performing tensions

Four performing tensions were identified (Table 6). Performing tensions become apparent when a variety of individuals or stakeholders pursue conflicting goals [22]. Our findings illustrate that innovation in healthcare is compromised when a multitude of parallel activities and tasks demand attention and resources. This was particularly evident in the tension between engagement in the Whole System Informing Self-management Engagement (WISE) project and annual planning by the Primary Care Trust (PCT) supporting the project, as described by Kennedy and colleagues. During implementation of their self-management support approach, the commitment of key stakeholders competed with the PCT’s long-term planning and resource allocation cycle [52].

**Table 6:** Performing tensions associated with healthcare innovation

Authors	Type of innovation	Stage of innovation process	Type of tension (dilemma or paradox)	Conflicting elements
Kennedy et al. (2014)	Self-managament support approach (WISE)	Implementing projects	Project engagement vs. Annual planning (Dilemma)	**A: Project engagement**: Commitment and buy-in from key stakeholders (p. 8) **B: Annual planning**: Existing long-term business and resource planning (p. 8)
Marjanovic et al. (2020)	Not specified	Implementing projects, Diffusing lessons	Cooperation vs. Competition (Dilemma)	**A: Cooperation**: Uptake of specific products, technologies, or new service and care models by connecting different stakeholders around the development (p. 292) **B: Competition**: Sociotechnical system and social fabric (e.g. ownership and accountability issues) challenge effective coordination (p. 292)
Mansfield et al. (2018)	Pilot project implementation in general	Implementing projects		**A: Cooperation**: Successfully engaging in pilot projects demands for collaboration (p. 9) **B: Competition**: A myriad of simultaneous pilot projects compete for access and resources (p. 10)
Vito (2017)	Improvement of access, accountability, evidence and coordination of children`s mental health services	Implementing projects		**A: Cooperation**: Valuing the idea of going through the process together and achieving a win win situation (p. 12) **B: Competition**: Competitive nature of the process leading to the demarcation between agencies (p. 12)
Shaller (2004)	Quality measures for children’s health care	Developing proposals, Implementing projects	Quality improvement vs. Implementation costs (Dilemma)	**A: Quality improvement**: Designing measures to improve quality of care (p. 219) **B: Implementation costs**: Need for a strong and compelling business case that clearly demonstrates the benefits of quality measurement relative to the costs of implementation (p. 222)
Sharp et al. (2020)	Smart phone application for use by rheumatoid arthritis patients	Implementing projects, Diffusing lessons	Clinical practice vs. User requirements (Dilemma)	**A: Clinical practice**: Practitioners’ preferences in regard to technological artifacts’ functionality and design has impact on implementation and diffusion (p. 7) **B: User requirements**: Patient needs must be taken into account in order to ensure adoption and diffusion of the technological artifact (p. 7)

The tension between cooperation and competition, a dilemma described a total of three times, also picks up on this. For example, Mansfield et al. describe physicians’ accounts of frontline tensions during the implementation of pilot projects to improve primary care: they observed a conflict between engagement in successful collaborative projects and further competing projects [53]. Marjanovic et al. point out the contradiction between the need to connect different stakeholders to improve care products or models and the requirements of existing social fabrics and systems [54]. Shaller describes tension between the implementation of targeted quality improvement measures for children`s healthcare and the need for a robust and compelling “business case” that justifies the costs of their implementation cost. He observed this dilemma during the “developing proposals” and “implementing projects” stages [55]. The fourth case of performing tension manifested as conflicting elements of clinical practice and user requirements. During the implementation of a smartphone app for rheumatoid arthritis patients, Sharp et al. found that there is a dilemma between the goals and requirements of clinical practice regarding the technology and design of the application and those of non-clinical users that needs to be negotiated to enable successful implementation and diffusion of the innovation [56]. We also grouped additional conflicting elements associated with performing tensions into the cross-cutting categories *performing/belonging* (Table 7) and *performing/organizing* (Table 8).

**Table 7:** Performing/belonging tensions associated with healthcare innovation

Authors	Type of innovation	Stage of innovation process	Type of tension (dilemma or paradox)	Conflicting elements
Oboirien et al. (2018)	District based clinical specialist team (DCST)	Implementing projects	Range of competence vs. Perceived authority (Dilemma)	**A: Range of competence**: DCST ought to organize, steer and support processes across the entire DHS structure (p. 7) **B: Perceived authority**: DCST perceived to be operating above the authority and jurisdiction of middle managers (p. 8)
Renju et al. (2010)	Young adolescent sexual reproductive health program	Implementing projects		**A: Range of competence**: Council HIV/ AIDS Coordinator (CHAC) ought to coordinate with comprehensive responsibility (p. 9) **B: Perceived authority**: CHACs perceived to be operating above their technical ability given their level of qualification and experience, thereby undermining the District AIDS Control Coordinator’s (DACC) authority (p. 9)
Leon et al. (2013)	Provider-initiated HIV testing and counselling (PITC)	Implementing projects	Provider-centeredness vs. Patient-centeredness (Dilemma)	**A: Provider-centeredness**: Taking an authoritative role of providing knowledge and advice as well as using a provider-centered communication style (p. 13) **B: Patient-centeredness**: Assessing patient readiness for testing and obtaining patient informed consent using a patient-centered form of communication (p. 13)

**Table 8:** Performing/organizing tensions associated with healthcare innovation

Authors	Type of innovation	Stage of innovation process	Type of tension (dilemma or paradox)	Conflicting elements
Marjanovic et al. (2020)	Not specified	Implementing projects, Diffusing lessons	System’s rigidity vs. System’s agility (Paradox)	**A: System’s rigidity**: Sociotechnical systems tend to be inward-looking and inert, propagating persistence and stability over change and innovation (p. 285) **B: System’s agility**: Innovation processes require system’s capacity to quickly and long lastingly adapt to change (p. 293)
Vito (2017)	Improvement of access, accountability, evidence, and coordination of children`s mental health services	Implementing projects	Accountability demands vs. Creating value (Dilemma)	**A: Accountability demands**: Ministry`s strategic directions increasingly focus on performance indicators and outcomes (p. 5) **B: Creating value**: The Ministry`s strategic directions focus on (…) service excellence, innovative leaders and cross-sectoral collaboration (p. 4)
Malik et al. (2017)	Linkages between human resource management (HRM) and innovation	Implementing projects	Exploration of new vs. Exploitation of existing (Dilemma)	**A: Exploration of new**: Looking for variation in existing routines (major or radical) to empower new products/services or to serve new markets and customers (p. 1359) **B: Exploitation of existing**: Seeking for improvements and refinements to the existing portfolio (p. 1359)
Valaitis et al. (2016)	Chronic disease prevention and sexually-transmitted infection prevention	Implementing projects	Local prerequisites vs. Evidence-based requirements (Dilemma)	**A: Local prerequisites**: Responding to local community needs and collaborative directions (p. 15) **B: Evidence-based requirements**: Providing health services that are grounded in evidence (p. 6 f.)
Valaitis et al. (2016)	Chronic disease prevention and sexually-transmitted infection prevention	Implementing projects	Legislative directions vs. Local accountability measures (Dilemma)	**A: Legislative directions**: Legislatively mandated requirements (p. 9) **B: Local accountability measures**: Accountability of the local board of health (p. 9)
Palmer et al. (2018)	Evidence-based episodes of care pathways as part of a hospital funding reform	Implementing projects	Standardizing cost vs. Standardizing care (Dilemma)	**A: Standardizing cost**: Addressing cost-pressures of quality-based procedures through standardization and scalability (p. 9) **B: Standardizing care**: Improving quality of care through the implementation of standardized clinical processes/care pathways (p. 9)

#### Belonging tensions

We identified five belonging tensions in the included studies (Table 9). Belonging tensions occur when competing stakeholder clash [22]. Our literature review, for example, highlights the conflict between prioritizing medical care and economic efficiency. In a study of innovations in the delivery and organization of endoscopy services in England and Wales, Rapport et al. observed a conflict between economic interests and medical convictions among health professionals in their focus groups. Regarding the question of which beliefs and values to prioritize in the implementation of healthcare innovations, they determined that it is important to decide whether to act based on the available resources or on medical evidence and needs [57]. We also observed different perspectives on innovation and related tensions within and between groups of healthcare stakeholders and professionals. Mansfield et al. highlight the significant role of identity, which manifests as tension between the individual and the collective: while stakeholders value their autonomy and status, the implementation of health services improvement initiatives requires inter-professional and multidisciplinary collaboration [53]. The findings of studies by Atun et al. and Lehn et al. point in the same direction [58, 59]. All three studies describe a dilemma arising from conflicts between professional ethics and self-perceptions of stakeholder groups and new collaboration requirements. The tension between patient illness experience and objectivation practices that accompanied innovations based on objectivation and scores was a theme highlighted in Sharp et al. [56]. This occurs when the convictions of patients diverge from those of the healthcare professionals treating them. Three sub-categories of tensions that overlap with belonging tensions only during project implementation were additionally identified. We classified these cross-category tensions as *learning/belonging* (Table 5), *belonging/organizing* (Table 10) and *performing/belonging* (Table 7).

**Table 9:** Belonging tensions associated with healthcare innovation

Authors	Type of innovation	Stage of innovation process	Type of tension (dilemma or paradox)	Conflicting elements
Rapport et al. (2010)	Endoscopy service delivery and organization	Implementing projects	Economic interest vs. Medical convictions (Dilemma)	**A: Economic interest**: Decision making based on availability of resources (passage of time, ability of staff members) (p. 926) **B: Medical convictions**: Demanding decisions based on medical evidence, patient need, or the immediacy of the problem (p. 926)
Mansfield et al. (2018)	Pilot project implementation in general	Implementing projects	Individual vs. Collective (Dilemma)	**A: Individual**: (Physicians, Clinicians) Valuing autonomy and independence, having control and routines (p. 8) **B: Collective**: Sharing data, improving local care coordination, and working interdisciplinary (p. 8)
Atun et al. (2007)	Family-medicine-centred PHC reform	Implementing projects	Professional boundaries vs. Interprofessional relations (Dilemma)	**A: Professional boundaries**: Traditional roles in PHC demarcate professional boundaries, thereby assuring a sense of authority, security, and quality of service (p. 34) **B: Interprofessional relations**: Expanding knowledge and skills through interprofessional relations, thereby stimulating confidence, performance, work efficiency and more control over professional duties (p. 34)
Lehn et al. (2018)	Readmission prevention program	Implementing projects	Interdisciplinary work vs. Professional autonomy (Dilemma)	**A: Interdisciplinary work**: Requirement for interdisciplinary cooperation in the initial screening stage (p. 8) **B: Professional autonomy**: Control and dependency on other professional groups leads to frustration and decreased motivation (p. 9)
Sharp et al. (2020)	Smart phone application for use by rheumatoid arthritis patients	Implementing projects	Illness experience vs. Objectivation practices (Dilemma)	**A: Illness experience**: Quantification gives way to an oversimplification of illness experience (p. 8) **B: Objectivation practices**: Relying on scoring systems and objective evidence to access treatment (p. 8)

**Table 10:** Belonging/organizing tensions associated with healthcare innovation

Authors	Type of innovation	Stage of innovation process	Type of tension (dilemma or paradox)	Conflicting elements
Shaller (2004)	Quality measures for children’s health care	Developing proposals, Implementing projects	Integration vs. Independence (Paradox)	**A: Integration**: Overcoming barriers and obstacles in concert with others and for other populations (p. 225 f.) **B: Independence**: Overcoming barriers and obstacles independently (p. 226)
Cresswell et al. (2016)	Technological innovation in healthcare	Identifying problems, Generating ideas, Developing proposals	Central coordination vs. Local collaboration (Dilemma)	**A: Central coordination**: Central coordination of innovative activity to spread knowledge and stimulate competition (p. 779) **B: Local collaboration**: Local collaboration to identify needs and build solutions based on these, creating safe spaces for collaborative endeavors (p. 779)

#### Organizing tensions

Generally speaking, organizing tensions arise when complex organizations generate competing designs and processes to achieve a desired outcome [22]. Organizing tensions associated with the complexity of healthcare organizations and of their activities and employees are listed in Table 11. Our findings reveal that the development, implementation, and diffusion of healthcare innovation creates tension between the need to simplify processes and standardize within the framework of complex structures and the need for flexibility and adaptability in order to meet the various (stakeholder) requirements and situations. Kreindler, Shaller, Hoekstra et al., Mignogna et al. and Sharp et al. describe dilemmas associated with tensions between standardization and fidelity and the need for customization and adaptability as some of the most significant organizational barriers to healthcare innovation [55, 56, 60–62]. Another study by Porter et al. described the dilemma surrounding the desire for standardization vs. autonomy [63]. The demands of healthcare and the goals of greater organizational standardization created an ongoing dilemma in these cases. Likewise, Hamilton et al., Mansfield et al., and Lehn et al. observed organizational tensions related primarily to dilemmas between adoption and implementation of innovation in certain forms of organizations, processes, or local situations [53, 59, 64]. Rowe and Hogarth, on the other hand, describe a paradox arising from the conflict between openness and standardization. They argue that an open process with the aim of high productivity requires openness, time for debate, and reflection, and that these goals conflict with the need for stability and mean higher levels of uncertainty during the change process [51]. This review revealed that organizing tensions show the greatest variety of tensions at different stages of the innovation process. Three sub-categories of tensions that overlap with organizing tensions were also identified. We classified these cross-category tensions as *learning/organizing* (Table 4), *belonging/organizing* (Table 10) and *performing/organizing* (Table 8).

**Table 11:** Organizing tensions associated with healthcare innovation

Authors	Type of innovation	Stage of innovation process	Type of tension (dilemma or paradox)	Conflicting elements
Hamilton et al. (2008)	Respiratory service	Developing proposals, Implementing projects	Central development vs. Local adoption (Dilemma)	**A: Central development**: Centrally driven innovation (p. 9) **B: Local adoption**: Local adoption of good ideas (p. 9)
Kreindler (2017)	Improvement of patient flow	Developing proposals, Implementing projects	Standardization vs. Customization (Dilemma)	**A: Standardization**: Spreading of best practice is impeded by an overemphasis of site uniqueness (p. 7) **B: Customization**: Spreading of best practice demands an allowance for site uniqueness (p. 7)
Mansfield et al. (2018)	Pilot project implementation in general	Implementing projects	Clinical duties vs. Project demands (Dilemma)	**A: Clinical duties**: Clinicians are required to provide patient care and not necessarily engage in research-related work (see quote p. 12). **B: Project demands**: Project trajectories require time and skills, i.e. for administrative responsibilities and research (p. 10)
Rowe and Hogarth (2005)	Four primary care trust (PCT) pilot sites	Implementing projects	Openness vs. Stability (Paradox)	**A: Openness**: Large productivity through an open process and need for openness and time for debate and reflection (p. 400) **B: Stability**: Levels of uncertainty during the process causing anxiety, sometimes expressed as hostility or distress (p. 400)
Shaller (2004)	Quality measures for children’s health care	Developing proposals, Implementing projects	Fidelity vs. Adaptability (Dilemma)	**A: Fidelity**: Need for standardization to assure comparability and consistency (p. 225) **B: Adaptability**: Preference to customize approaches to fit circumstances or at least retain the option to do so (p. 225)
Hoekstra et al. (2017)	Health promotion program in multi-disciplinary settings	Implementing projects		**A: Fidelity**: Implementation of a program according to protocol (p. 2) **B: Adaptability**: Adapting a program to the local context (p. 2)
Mignogna et al. (2018)	Cognitive behavioral therapy in primary care	Implementing projects		**A: Fidelity**: Delivering of a treatment as it was intended to be delivered (p. 2) **B: Adaptability**: Changing the intervention to improve its “fit” (p. 2)
Sharp et al. (2020)	Smart phone application for use by rheumatoid arthritis patients	Implementing projects, Diffusing lessons		**A: Fidelity**: Widening the use through standardization and efficiency (p. 12) **B: Adaptability**: Meeting specific patient groups and diagnostic needs (p. 12)
Lehn et al. (2018)	Readmission prevention program	Implementing projects	Interdisciplinary work vs. Mono-professional working structures (Dilemma)	**A: Interdisciplinary work**: Requirement for interdisciplinary cooperation in the initial screening stage (p. 8) **B: Mono-professional working structures**: Information and demand for interdisciplinary responsibilities and judgment is unclear and in opposition to the accepted organization of clinical work (p. 8)
Porter et al. (2018)	Computerised clinical decision support (CCDS) in emergeny pre-hospital care	Implementing projects	Standardization vs. Autonomy (Dilemma)	**A: Standardization**: Professionalizing paramedic practice by using formalizing and standardizing tools (p. 8) **B: Autonomy**: Autonomy of clinical decision-making as part of professionalism in paramedic practice (p. 8)

### Cross-category tensions

Cross-category tensions are those that operate between two main categories of tension. In the following section, we present the findings of studies in the literature describing such cross-category tensions.

#### Learning/organizing tensions

Two studies reported tensions operating between the categories of learning and organizing (Table 4). These cross-category tensions arise when routines aimed at stability are at odds with the need for speed and flexibility [22]. In an article on the implementation of non-invasive prenatal testing for aneuploidy in a national healthcare system, van Schendel et al. illustrate the dilemma surrounding the new need for collaboration to achieve benefit from the innovation versus the loss of stability and focus due of new standardized digital communication tools to speed up patient enrollment led to a to the shift in work tasks and responsibilities [65]. Lehn et al. described how the use conflict between established tools and underlying strategies [59]. Both cases represent dilemmas that must be resolved in order to create an agile and effective organization. The example of Lehn et al. also shows that the same tensions can occur in different contexts. Thus, tensions related to standardization could also be located in the categories of *organizing* (Table 11) and *learning/belonging* (Table 5).

#### Learning/belonging tensions

Table 5 summarizes the seven tensions between learning and belonging identified here. Learning/belonging tensions arise when a person’s developed sense of self and purpose and the desire to hold on to it collide with a situation that requires adaptation and change [22]. Lehn et al. describe an example in which the use of a standardized approach to patient enrollment in a prevention program conflicted with healthcare professionals’ self-conception that they were competent to identify program candidates based on their expert judgment [59]. Professionalism of NHS staff and its respective biomedical understanding is also described as conflicting with an alternative, more contextual and psychological view of a therapeutic intervention. The fundamental discrepancy in the views of the practitioners described by Mathers et al. represents a paradoxical tension [66]. The innovative use of telemedicine in healthcare was also identified as a potential source of conflict. Gagnon et al. describe a case in which tension arose between a regionally established referral process and a new service involving non-regional care specialists [67]. Bagot et al. point out the tension between specialists` lack of trust in the skills of less specialized clinicians and increased access to treatment through their involvement in a telemedical collaboration [68]. Forms of partnership or collaboration were repeatedly identified as opposing elements of routine preservation. Bonello et al. described an example in which this occurred in interprofessional education and Merkel et al., who evaluated the implementation of a new treatment process, illustrated how shifting from established norms and responsibilities to more cooperative approaches create tensions [69, 70]. Besides routine preservation, a focus on core tasks may also conflict with the motivation for change or drive for improvement, as was reported by Gardner et al. [71].

#### Belonging/organizing tensions

Two studies identified in this review reported tensions between belonging and organizing (Table 10), which occur when tensions between the properties and characteristics of the individual and the collective emerge [22]. Shaller describes how such tensions occurred during the implementation of new quality measures for children’s healthcare when the overcoming of challenges by the individual conflicted with the overcoming of challenges by an integrated group of people. Integration and independence are conflicting elements that arose during the implementation and development of the innovation project [55]. We defined this example as a paradox because the perception of self as an individual in this case precludes belonging to a group and, thus, precludes an integrative approach to resolution. Cresswell et al. describe a case where conflict between central and local coordination of a technological innovation arose during several phases of the innovation process [72]. This case is another dilemma that clearly illustrates how individual and collaborative conceptions of needs and success factors should be balanced during the development of healthcare innovations.

#### Performing/belonging tensions

Tensions between performing and belonging arise when individual identities clash with social and occupational demands [22]. Performing/belonging tensions were identified in three studies reviewed here. This type of conflict between stakeholders’ identities and goals manifests as the conflicting elements of range of competence and perceived authority. It occurred as a dilemma in the following two studies: Oboirien et al., who evaluated the implementation of district-based clinical specialist teams in South Africa, show how performing/belonging tensions associated with changing role requirements and functions in the context of innovation can lead to role ambiguity and conflict [73]. Renju et al. observed the same conflict in the introduction of a health program where the stakeholders’ new responsibilities were in conflict with their qualifications and experience [74]. The performing/belonging tensions identified in Leon et al. can be classified as a dilemma, too: These investigators show that although seemingly contradictory, both an authoritative, advisory style of communication and a patient-centered, demand-oriented form of communication are needed in the same innovative approach [75]. Because the seemingly paradoxical conflict between the conventional provider-centered and the new patient-centered communication style can potentially be resolved, for example, by a patient guideline with latitude for patients, but which is implemented and executed very strictly, we classified this as a dilemma. Table 7 summarizes these three cases of performing/belonging tensions.

### Performing/organizing tensions

We identified a total of six cases of performing/organizing tensions (Table 8). “*Tensions between organizing and performing can be summarized by the interplay between means and ends*” [22 p383]. According to Marjanovic et al., paradoxical tensions may surface as a result of conflict between a sociotechnical system’s rigidity and tendency to value stability over innovation and a health system’s need for agility to adapt to change [54]. The conflict between the desire to create value through the further development of a unit, procedure or routine and the simultaneous preservation of established requirements and outcome expectations was also described by Vito and Malik et al. [76, 77]. In addition, two cases of tension between general, central demands and those of local customers were described in Valaitis et al.: The first occurred during the implementation of a prevention program, when local community prerequisites conflicted with evidence-based requirements for health services. In the second case, legislative requirements and the accountability of the local board of health represented contradictory positions that led to a dilemma [78]. Palmer et al. describe tension resulting from the dilemma of standardizing costs versus standardizing care during the implementation of an innovative episode of care pathway. The aim of the hospital funding reform evaluated by these investigators was to improve the quality of care by standardizing care processes and pathways while at the same time addressing the cost pressure from the implementation of quality-based procedures [79].

#### Management strategies used in healthcare innovation

Fourteen of the included studies investigated management strategies to mitigate, resolve, or enhance the creative potential of the multi-layered tensions and conflicts encountered in healthcare innovation processes (Table 12). “Both/and” strategies were used in 12 cases, and “either/or” strategies in the remaining two. Both/and strategies aim to achieve dynamic equilibrium or balance between two opposing poles of tension by addressing both poles and shifting between, integrating and/or balancing them [13, 22]. Either/or approaches seek to promote a trade-off between or a separation of contradictory demands and ask, “Under what conditions would I choose A or B?” [15]. More-than strategies, whose goal is to shape a new perspective or create a new element that exceeds the existing elements, were not mentioned in any of the studies included here. This may be a consequence of the complexity implementing such solutions and the concomitant failure to apply them as well as the resulting lack of reports. However, future research should pay more attention to these approaches, as they can leverage the creative, synergistic potential of tensions and generate virtuous cycles that drive innovation in healthcare systems [13].

**Table 12:** Management strategies used in healthcare innovation

Authors	Tension	Strategy type	Strategy description
Cresswell et al. (2016)	Central coordination vs. Local collaboration	Both/and	Creating safe spaces for collaboration, incentives to encourage innovative activities, innovative funding models, and a climate that allows for experimentation and possible “failure“ (p. 779)
Rowe and Hogarth (2005)	External facilitation vs. Internal satisfaction	Both/and	Combining the change of external organizational attractors with the facilitation of exposure and debate to enable professionals and leaders to recognize and work toward future externally imposed models of health care through the lens of their own fundamental attractor patterns (p. 403)
Merkel et al. (2015)	Routine preservation vs. Interprofessional relations	Both/and	Moderation between poles and monitoring of conflicts about competences and responsibilities (p. 7)
Van Schendel et al. (2017)	Collaboration vs. Shifted tasks/responsibilities	Either/or	Conducting regular meetings to create better cooperation, close cooperation and stimulating mutual understanding between stakeholders (p. 8)
Hamilton et al. (2008)	Central development vs. Local adoption	Both/and	Identifying models that offer advantages to clinicians and managers (p. 9)
Kreindler (2017)	Standardization vs. Customization	Both/and	Establishing a coherent process based on the analysis of the entire trajectory of care of a patient population (p. 13)
Hoekstra et al. (2017)	Fidelity vs. Adaptability	Both/and	Identifying pre-defined ‘core components’ of the program that are needed to be implemented strictly according to the protocol while allowing a flexible implementation of the ‘adaptable elements’ of the program (p. 2)
Marjanovic et al. (2020)	Cooperation vs. Competition	Either/or	Strengthening cooperation by placing cross-organizational stakeholders in management positions (p. 292)
Shaller (2004)	Quality improvement vs. Implementation costs	Both/and	Building a sustainable business case that is based on a cost-benefit analysis and includes evidence that the intervention is saving (direct) costs of care as well as improving quality and non-monetary factors (p. 224)
Sharp et al. (2020)	Clinical practice vs. User requirements	Both/and	Mediating different user requirements through compromise in app design (p. 7)
Vito (2017)	Cooperation vs. Competition	Both/and	Collective Leadership that gives space for different roles and expectations and develops solutions collaboratively (p. 22)
	Accountability demands vs. Creating value	Both/and	Adaptive leadership that fosters creativity and ideas among staff and leads with vision, flexibility and tolerance for risks and insecurities (p. 7)
Oboirien et al. (2018)	Range of competence vs. Perceived authority	Both/and	Facilitating role adaptation and integration into the health system through mentors that provide ongoing support (individual and institutional) (p. 10)
Malik et al. (2017)	Exploration of new vs. Exploitation of existing	Both/and	Facilitating of ambidexterity through changes in staff contextual conditions (e.g. hiring local (and regional) talent, developing performance management metrics for supervisors to facilitate the generation of new ideas, mentoring of frontline staff, flexible benefits, and training) (p. 1374)

## Discussion

Tensions were identified and described in various studies discussing greatly heterogeneous types of healthcare innovations and settings. Focusing closely on tensions has proven fruitful to go beyond simply addressing instances of conflicts (i.e. barriers) and to instead acknowledge the complex courses of healthcare innovation processes. The findings point to the following underlying factors that typically fuel conflicts during innovation processes in healthcare systems: (a) willingness to innovate against the backdrop of standardized specifications, medical convictions and liability issues, (b) established internal and external environments as well as stakeholder roles and expectations such as autonomy and accountability, and (c) lived and ascribed role characteristics, hierarchies and beliefs that are reproduced and manifested through education, work environment, and societal views. Besides inertia of the structures, one reason for this may be differences in the working cultures involved and friction between them [5]. Yet, tensions in innovation implementation arise not only due to inter-professional cooperation, but also because different professional groups with their own hierarchies have to work together [80]. It also can be assumed that escalating commitments within these fields account for a certain proportion of the tensions that occur. The importance of medical sectors, professions, or working cultures, and the boundaries between them, in triggering and accumulating tensions should therefore be examined more closely. This differentiated view would allow further recognition of the role of institutional framing in the emergence of not singularly occurring conflicts between stakeholders. This is supported by the findings of several reviewed studies, which show that digital innovations such as innovative telemedicine-based care concepts cause tensions [63, 68]. The conflicts are driven by the associated changes in organizations, forms of work, and areas of responsibility associated with digital innovations. Assuming that innovations are currently driven by the possibilities of digitization, the mitigation of these conflicts should therefore be addressed. This can succeed due to the fact that the great majority of tensions described in the reviewed literature represent a dilemma, hence conflicts can be managed and resolved. The perception of paradox is likely to vary among the stakeholders involved. Although the line between paradox and dilemma is not always clear-cut, irresolvable contradictions are the absolute exception and true paradoxes are very rare [43]. This is because the views of stakeholders as well as legal and financial frameworks can be negotiated and adjusted as needed. This suggests great mitigation potential and provides opportunities for effective innovation support.

Appropriate management activities represent one possibility for this support. Because it takes time to negotiate and overcome conflicts, innovation projects should be accompanied by change management which proactively analyzes typical tensions in advance, avoids or at least limits them, and secures flexible potential in order to be able to (re)act on unforeseen tensions/crises in the course of the innovation. That this can be successful has been described by Bagot et al. in whose case a reduction of tensions was achieved and even maintained after implementation [68]. As shown in Fig. 3, most conflicts reported in the studies included in our analysis occurred mainly in the process stage of implementation. Management activities should therefore be carried out especially in this process phase. Reflected tensions management could push forward the whole process and the right timing for interventions is an important part of the strategy here. This temporal component should be taken into account when looking at the process-related course of an innovation. Important processes in the overall context of change and linkage mechanisms, interdependencies, and temporal sequences across multiple levels of analysis are extremely insightful.

On the macro level, overarching, deep-seated systemic conflicts must be identified to effectively address tensions and explore potentials for innovation. Reflection on challenges on that level can also help to overcome predefined paths and enable the constructive potential of tension-induced changes in direction of the innovation process. Tension can then be less of a threat and more of a positive impetus to break out of the system`s inherent dependencies and pursue constructive trajectories. Using UK healthcare as an example, Cresswell et al. come to similar conclusions and highlight the potential effects of actions on system level in addition to the individual level to create a climate for innovation [72]. On meso and micro level it is important to develop a better understanding of the importance of small events in the creation, detection, and amplification of tension. Since the course of innovation processes cannot be completely planned, there are not only tensions that can be avoided or exploited through better planning and avoidance of management failures, but also tensions that cannot be predicted and must be addressed when they occur [8, 81, 82]. This again requires sensitivity to random and small outcomes as well as flexibility and short-term planning, for example in the form of agile project management or reflexive change management. In addition to long-term planning, this reflexivity further increases the avoidance and effective management of tensions.

This provides a good starting point for further research on the reasons and context for the emergence of tensions and how tensions are managed. Moreover, the internal and external frameworks of these interactions must be studied with an appropriately process-sensitive method that also considers sub-processes at different levels as the basis for developing innovative ideas in healthcare. Further research should also focus on decision-making processes within the organization and the specific but overarching organizational culture and its effects on the adaptation, implementation, and diffusion of innovation [83]. Original empirical research has proven its worth as a method for surveying these factors and general conditions. Qualitative methods can be used to build on the insights gained in previous studies. These research approaches should be used to examine interactions between the aforementioned factors directly and to further our understanding of tensions in healthcare innovation as well as to analyze how and why certain events and actions of individual actors unfold over time. This can be done, for instance, according to the example of Mintzberg [84].

### Limitations

The findings of this review are subject to some limitations. First, it is important to note that tensions, conflicts, and related terms are rarely used directly but only implicitly, making them difficult to identify in the literature. As the research question is characterized by a high degree of specificity, it was necessary to conduct additional manual and backward literature searches to identify articles for the present review. Furthermore, tensions that lead to the termination of projects or to fundamental changes of project setting are probably underrepresented in the literature. This may be a reason why we predominantly identified tensions occurring in the project implementation phase. Therefore, it is likely that studies and reports of projects or innovations that were discontinued or modified prior to implementation were not published. Tensions from the first process steps or from discontinued projects usually are not reported or considered in the literature. This also applies to the findings from healthcare systems analyses. The fact that many studies included in this review report observations from government-funded healthcare systems may be due to the fact that those are the types of systems that we report most often. Further research is needed to address these issues and to explore which types of healthcare systems are conducive to the emergence and management of innovations in order to provide insights on the successful implementation of innovations in healthcare systems. For this, the level of data analysis must also be questioned, and the macro level must be brought more into focus. The discourse in current tensions research, and the literature on healthcare innovation, generally focuses on the micro level. This became also apparent in our review, with the majority of authors describing tensions at the micro level (see e.g. Kennedy et al. and Rowe and Hogarth [51, 52]). The description of tensions on the meso or macro level as it is done for example in the works of Vito et al. and Marjanovic et al. is the exception [54, 77]. However, it can be assumed that important factors exist at the system level regarding the emergence of tensions. Since few studies describe conflicts at this level, this deficit should be addressed, and the macro perspective should be more strongly addressed in future considerations.

## Conclusion

In this systematic literature review, we identified nine categories of tensions typically associated with the implementation of innovation in healthcare systems and additionally described conflicting elements of the innovation process. Our research contributes to the literature on innovation in healthcare by describing the typical types of tensions associated with healthcare innovation, identifying the types of innovations in which they occur, and demonstrating how they are interrelated. In summary, the various requirements, working cultures, and values of stakeholders as well as the framework of complex structures and standards within a given healthcare systems are factors that potentially trigger tensions. The accumulation of tensions in government-funded healthcare systems suggests that such systemic structures foster the emergence of conflicts. The fact that the conflict between centralized, legislator-determined financial and organizational frameworks and design and organizational requirements at the micro or meso level was a recurring element in healthcare innovation across different countries and health systems supports this notion [56, 60]. Furthermore, we used a process view to analyze the innovations described in the literature. The insights gained with this description and classification approach provide a better understanding of the underlying causes of tensions, for example, regarding the influenceability, extent and strength of relevant interactions in innovation processes. Avenues for future research include the (regulatory) improvement of innovation conditions and coping and management strategies that significantly influence the successful management of tensions and thus have the potential to improve the control and outcome of healthcare innovation initiatives. Studying and analyzing tensions in selected areas of healthcare innovation can help to develop a better, contextual understanding of the complexity of innovation processes.

## Data Availability

The datasets used and/or analyzed during the current study are available from the corresponding author on reasonable request.
